# A new method of N to C sequential ligation using thioacid capture ligation and native chemical ligation

**DOI:** 10.1098/rsos.172455

**Published:** 2018-06-20

**Authors:** Wen Hou, Lei Liu, Xiaohong Zhang, Chuanfa Liu

**Affiliations:** 1 First Center Hospital Clinic Institute, Tianjin Medical University, Tianjin 300192, China; 2 Key Laboratory for Critical Care Medicine of the Ministry of Health, Tianjin First Center Hospital, Tianjin 300192, China; 3 Organ Transplant Center, Tianjin First Center Hospital, Tianjin 300071, China; 4 Division of Chemical Biology and Biotechnology, School of Biological Sciences, Nanyang Technological University, 60 Nanyang Drive, Singapore 657551, Republic of Singapore

**Keywords:** sequential peptide ligation, thioacid capture ligation, native chemical ligation, protein chemical synthesis

## Abstract

Sequential peptide ligation strategy becomes more and more important in large protein or long peptides chemical synthesis due to the limited peptide/protein size obtained by solid phase synthesis of individual peptides or even one-step peptide ligation. Herein, we developed an alternative method which could perform the sequential peptide ligation of several segments from N to C direction based on the combined use of thioacid capture ligation and native chemical ligation. The sweet protein monellin was produced through this strategy on a scale of multi-milligrams.

## 1. Introduction

Chemical synthesis is playing a more and more important role in protein production, especially after Merrifield's innovation of solid phase peptide synthesis (SPPS) in 1963 [1]. Nevertheless, it is not easy to get homogeneous long peptides through SPPS. The chemical ligation techniques (for reviews, see [2–9]) have significantly expanded the size limit of SPPS. However, the products formed by ligation of two segments are still smaller than majority of natural proteins. Although protein semi-synthesis [10,11] can be used where one segment can be prepared by recombinant methods to synthesize full-length proteins, the size of the other segment of the synthetic peptide is still a restriction. Considering that typical protein molecules in nature consist of approximately 300 amino acids, the conduction of sequential or convergent ligation of multiple segments to synthesize proteins is necessary. Sequential peptide ligations can be done from C-to-N and N-to-C directions. At the beginning, most sequential peptide ligations were based on the C-to-N strategy using native chemical ligation, but the N-terminal cysteine residue of the internal thioester segments must be protected to prevent undesired self-ligation or cyclization reaction [12]. To decrease the multiple intermediate purification and lyophilization steps, one-pot ligation method was developed [13–18]. While the sequential ligation from C to N direction is straightforward, the N-to-C strategies including two or more orthogonal ligation methods have attracted more attention recently [16,19–28]. The keystone of these strategies is that the C-terminal moiety of the internal cysteinyl peptide should remain intact during the first ligation and therefore will be available for the following ligation steps. Previously, our laboratory introduced an N-to-C sequential ligation approach [24] based on the differential reactivity between a regular peptidyl thioester and *N,N*-bis(2-mercaptoethyl)amide which was developed by our group [29] and Melnyk's group [30].

Herein, we developed another new method that enabled the sequential peptide ligation from N to C direction based on two ligation methods, thioacid capture ligation [31] and native chemical ligation [32]. In the thioacid capture ligation, a peptide thioacid reacts with the 2-mercapto-5-nitropyridyl (Npys) activated cysteinyl peptide to form a new peptide with Cys as the ligation site under acid condition. Nevertheless, in native chemical ligation, a peptide thioester and a cysteinyl peptide are ligated together under mild base condition. Based on these two orthogonal ligation methods, we designed a central peptide with a thioester at the C terminal and an Npys protected Cys at the N terminal. This Npys protected cysteinyl peptide thioester could ligate to a peptide thioacid by thioacid capture ligation efficiently without no self-ligation and cyclization under the acid condition. Meanwhile the thioester moiety of the central peptide is available for the subsequent native chemical ligation.

**Scheme 1.**
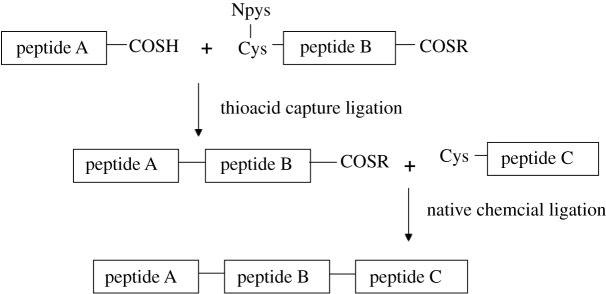
Strategy for synthesis single-chain monellin from N to C direction.

To test our proposal, we synthesized the sweet protein monellin as a demonstration. Natural monellin is a sweet protein isolated from the fruit of the tropical plant *Dioscoreophyllum cumminsii* Diels [33]. It is 3000 times sweeter than sucrose on a weight basis [34]. Monellin consists of two polypeptide chains, chain A and chain B. Chain A contains 44 amino acids, and chain B contains 50 amino acids. A single chain monellin (MNEI) containing 96 amino acids was designed by joining the B and A chains via Gly-Phe dipeptide [35]. This single chain monellin is more stable under extreme heat and pH conditions without changing its sweetness. We demonstrated our N-to-C sequential ligation strategy by synthesizing this single chain monellin (scheme 1). The protein was divided into three segments, MNEI(1-40) thioacid (peptide A), MNEI(41-65) thioester with Npys protecting group at the thiol group of Cys^41^ (peptide B), and MNEI(66-96) (peptide C). The 66th residue Ala was changed to Cys as the ligation site.

The whole sequence of MNEI is as follows:

GEWEIIDIGPFTQNLGKFAVDEENKIGQYGRLTFNKVIRP^40^-CMKKTIYEEN REIKGYEYQLGFYVY^65^-ASDKLFRADISEDYKTRGRKLLRFNGPVPPP^96^. The underlined residue is changed to Cys to accommodate ligation at this site.

## 2. Material and methods

### 2.1. Materials

All amino acids and coupling reagents were purchased from GL Biochem (Shanghai, China) and Novabiochem (Germany). MBHA and Wang resins were purchased from GL Biochem (Shanghai, China). All chemical reagents were purchased from Sigma.

### 2.2. High-performance liquid chromatography analysis and purification

Analytical and semi-prep high-performance liquid chromatography (HPLC) were performed on a Shimazu HPLC system equipped with a SPD-M20A prominence diode array detector. A C18 reversed-phase column (Jupiter 5 µm C18 300 Å, 250 × 4.6 mm) was used for analytical analysis. A C18 reversed-phase column (Jupiter 5 µm C18 300 Å, 250 × 10 mm) was used for semi-prep purification. The analytes were eluted using a gradient mixture of two solvents: solvent A was distilled deionized water containing 0.05% trifluoroacetic acid (TFA) and solvent B was 90% acetonitrile (ACN) in distilled water containing 0.05% TFA. The mobile phase flow rate was 1 ml min^−1^ for analytical analysis and 2.5 ml min^−1^ for semi-prep purification. The separation temperature was room temperature.

### 2.3. Mass spectrometry

Electrospray ionization (ESI) mass spectra were recorded using a bench-top ion trap mass spectrometer (FINNIGAN LCQ Deca XP MAX) equipped with standard ESI sources.

Mass of protein was recorded on a 4800 MALDI TOF/TOF Analyzer from Applied Biosystems which uses tandem time-of-flight technology for tandem mass spectrometry and is designed for sensitive, high throughput protein identification. The mass spectra were obtained in the MS reflector positive ion mode and using sinapic acid as the matrix.

### 2.4. Synthesis of peptide A thioacid

The peptide thioacid was produced by a solid-phase hydrothiolysis method which was developed by our laboratory previously [36]. First, thio-functionalized ChemMatrix (CM) resin was prepared by coupling Trt-SCH_2_CH_2_COOH onto commercial aminomethyl CM resin followed by detritylation with 5% TFA. Peptide was then assembled on the thiol-derived resin using typical Boc-chemistry SPPS protocols. All amino acids were used in 4 eq of the resin, and preactivated by 4 eq of PyBop and 8 eq of diisopropylethylamine (DIEA). After 1 h coupling of the first amino acid residue, the resin was washed with dichloromethane (DCM; 3×), dimethylformamide (DMF; 3×) and DCM (3×). Capping was performed by using 5% acetic anhydride/2.5% DIEA in DMF/DCM (1:1) for 2 × 10 min. The resin was washed with DCM (3×), DMF (3×) and DCM (3×). After capping, the successive α-amino group deprotection steps were performed in 30% TFA/DCM 2 × 10 min between each amino acid coupling step. The following side chain protecting groups were used for peptide A: Mts for Arg, 2-Cl-z for Lys, Xan for Asn and Gln, OBzl for Asp and Glu, Bzl for Thr and Tyr. Coupling was conducted using threefold excess of amino acids and PyBop in DCM. Wash steps were carried out with DCM and DMF. The successive deprotection of Boc on *α*-amino group was performed in 30% TFA in DCM. Qualitative ninhydrin test [37] was used to monitor the completeness of the coupling. In the case of Arg(Mts), double coupling had to be done. Final deprotection was conducted by using a cocktail of trifluoromethanesulfonic acid:thioanisole:TFA/1:2.5:10 at room temperature for 1 h.

After washing by TFA and ACN, the deprotected peptides bound on the resin were released from the resin by hydrothiolysis by using 0.1 M Na_2_S or 0.2 M (NH_4_)_2_S in 0.3 M HEPES buffer, pH 8.6 at room temperature. The cleaved peptide thioacid was analysed by analytical C18 RP-HPLC column and purified by semi-prep C18 RP-HPLC. Molecular weight of the peptide thioacid was measured by ESI-MS.

The sequence of peptide A thioaid is H-**GEWEIIDIGPFTQNLGKFAVDEEN-KIGQYGRLTFNKVIRP**-SH.

### 2.5. Synthesis of peptide B thioester

Peptide B thioester was synthesized by manual SPPS Boc chemistry using MBHA resin. First of all, the thio-functionalized MBHA resin was prepared by coupling Trt-SCH_2_CH_2_COOH directly to the MBHA resin. Trityl group was removed by 5% TFA three times, and detritylation was confirmed by Ellman test. Then peptides were assembled on the thiol-derived resin using typical SPPS protocols.

The sequence of peptide B is H-**CMKKTIYEENGFREIKGYEYQLYVY**-SCH_2_CH_2_CONH_2._ The following side chain protecting groups were used for peptide B: Bzl for Thr and Tyr, OcHx for Glu, Trt for Asn and Gln, Tos for Arg, 2-Cl-z for Lys, 4-MeOBzl for Cys.

After synthesis of the whole peptide sequence and deprotection of the N terminal amino acid residue, the resin was dried and applied to standard HF cleavage procedure. First of all, the resin, a stirring bar and scavenger mixture (*p*-cresol/anisole 1:1) were placed to a HF reaction vessel. Secondly, the vessel was frozen by liquid nitrogen and vacuumed. Thirdly, HF was transferred to the vessel by 10 volume of *p*-cresol or anisole. The mixture was conducted at 0°C for 1 hour. Fourthly, HF was evaporated and the peptide was precipitated by diethyl ether. Finally, all the solution and precipitate were transferred to a Büchner funnel, and the peptide was eluted by ACN/H_2_O (1 : 1). The crude product was lyophilized and purified by HPLC.

### 2.6. Protection of N-terminal Cys by Npys of peptide B thioester

5 eq. 2,2′-dithiobis(5-nitropyridin) and 1 eq. peptide B thioester were dissolved in acetic acid. The mixture was shaken for 6 h at room temperature. The product was confirmed by ESI-MS, and purified by HPLC and lyophilized.

### 2.7. Synthesis of peptide C

Peptide C was synthesized using Fmoc chemistry on Wang resin. Firstly, 4 eq. Fmoc-Pro-OH of the resin and 4 eq. DIC were dissolved in dry DMF/DCM, and reacted for 10 min. Secondly, 0.4 eq. DMAP was added and all the solution was transferred to the resin in a reaction vessel. The loading reaction was allowed overnight. Finally, the washed resin was loaded to a microwave peptide synthesizer to synthesize peptide from the second residue of the peptide from C terminal to N terminal. The sequence of peptide C is H-**CSDKLFRADISEDYKTRGRKLL-RFNGPVPPP**-OH. The following side chain protecting groups were used for peptide C: Trt for Cys and Asn, tBu for Ser, Thr and Tyr, OtBu for Asp and Glu, Boc for Lys, Pbf for Arg.

After deprotection of the N terminal residue of peptide C, the resin was dried and cleaved by 95% TFA, 2% H_2_O, 1.5% Tis, 1.5% 2-mercaptoethanol in volume for 3 h. The peptide was precipitated by ethyl ether, and then lyophilized and purified by HPLC.

### 2.8. Ligation of peptide A thioacid and Npys-peptide B thioester

1 eq. peptide A thioacid (final concentration 5 mM) and 2 eq. Npys-peptide B (final concentration 10 mM) were added to a 6 M Gdn.HCl and 100 mM acetate buffer (pH 3.0). 10 min later, the pH was adjusted to 6 by phosphate buffer. After incubation at 37°C for 2 h, 10 eq. tris(2-carboxyethyl)phosphine (TCEP) was added to give a reductive environment, and then the reaction was monitored by HPLC. The ligation product was confirmed by ESI-MS and purified by HPLC.

### 2.9. Native chemical ligation

The ligation product A + B (final concentration 5 mM) and peptide C (final concentration 10 mM) were added to 20 mM TCEP, 6 M Gdn.HCl 200 mM Hepes buffer (pH 8.0). Then 2% (w/v) thiophenol and 2% (w/v) sodium methanethiolate were added. pH was adjusted to 8.5. The reaction mixture was incubated at 37°C for 48 h. The reaction was monitored by HPLC. The final ligation product was confirmed by ESI-MS and purified by HPLC.

### 2.10. Circular dichroism

Steady-state circular dichroism (CD) spectra were measured in the far-UV light range (185–260 nm) using a Chirascan spectropolarimeter. Spectra were recorded in a 60 µl quartz cell (Hellma) with a path length of 0.1 mm, at 20°C and a step resolution of 1 nm. The readings were the average of 1 s at each wavelength, and the recorded ellipticity values were the average of three determinations for each sample. CD spectroscopy of the protein (2.2 mg ml^−1^) was performed in a buffer of 50 mM phosphate (pH 7). The average of three buffer spectra were subtracted from the averaged spectrum of the protein. CD values were converted to mean residue ellipticity (Q) in units of deg cm^2^ dmol^−1^ using the software Chirascan version 1.4 (Applied Photophysics). This baseline-corrected spectrum was used as input for computer methods to obtain predictions of secondary structure.

## 3. Results

### 3.1. Synthesis of peptide A thioacid

**Figure 1.**
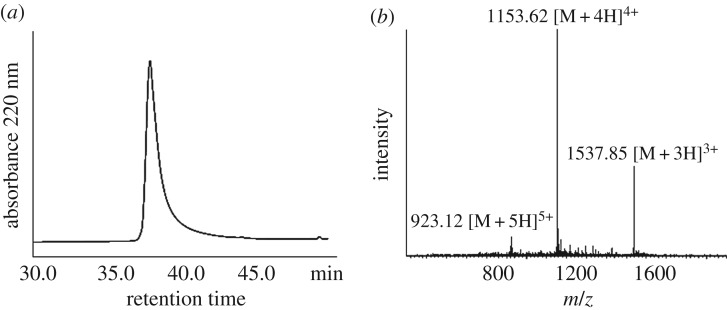
Characterization of peptide A thioacid. (*a*) C18 analytical HPLC profile of peptide A thioacid. HPLC condition: 0% to 20% of solvent B in solvent A in 5 min, followed by 20%–60% of solvent B in solvent A in 40 min. (*b*) Mass spectrum of this peptide determined by ESI-MS. [M + 3H]^3+^ found: 1537.85, MW calcd: 4610.5.

We generated peptide A thioacid, with the sequence of H-**GEWEIIDIGPFTQN- LGKFAVDEENKIGQYGRLTFNKVIRP**-SH, on CM resin. CM resin has a high degree of cross-linking and exhibits good loading capacity and mechanical stability. Most importantly, it is well-solvated in a broad range of nonpolar and polar solvents, including water. This makes it an excellent candidate resin for solid phase-supported reactions in aqueous media. We used the CM resin for the synthesis of peptide thioacids through hydrothiolysis of resin-bound peptide thioesters. Either ammonium sulfide or sodium sulfide was used as the source of hydrosulfide ions. The deprotected peptide thioester resin was suspended in hydrothiolysis buffer in an Eppendorf vial with gentle shaking. The reaction was stopped by acidification with 20% TFA/H_2_O on ice cooling. The mixture was subject to HPLC purification to give the purified thioacid peptide, and the molecular weight of peptide thioacid was determined by ESI-MS (figure 1).

### 3.2. Synthesis of peptide B thioester

**Figure 2.**
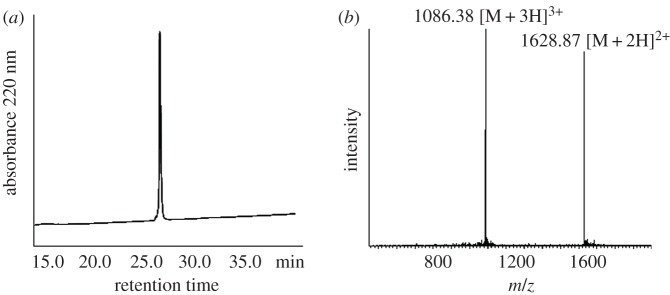
Characterization of peptide B thioester. (*a*) C18 analytical HPLC profile of peptide B thioester. HPLC condition: 0% to 20% of solvent B in solvent A in 5 min, followed by 20%–60% of solvent B in solvent A in 40 min. (*b*) Mass spectrum of this peptide determined by ESI-MS. [M + 2H]^2+^ found: 1628.87, MW calcd: 3255.

Peptide B thioester, with the sequence of H-**CMKKTIYEENREIK- GYEYQLGFYVY**-SH was synthesized by standard Boc SPPS. The thioester bond was directly generated on the MBHA resin by using S-trityl mercaptopropionic acid. The amino acids were assembled onto the mercaptopropionyl MBHA resin following standard SPPS protocols. After HF deprotection and cleavage, the peptide thioester was released from the resin. The peptide thioester was purified by RP-HPLC and the molecular weight was determined by ESI-MS (figure 2).

### 3.3. Converting peptide B thioester to Npys-peptide B thioester

**Figure 3.**
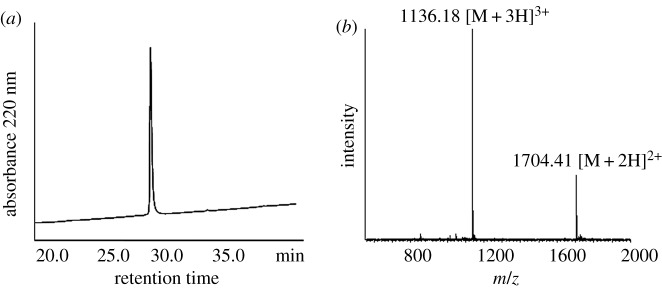
Characterization of Npys-peptide B thioester. (*a*) C18 analytical HPLC profile of Npys-peptide B thioester. HPLC condition: 0% to 20% of solvent B in solvent A in 5 min, followed by 20%–60% of solvent B in solvent A in 40 min. (*b*) Mass spectrum of this peptide determined by ESI-MS. [M + 2H]^2+^ found: 1704.41, MW calcd: 3409.

After purifying the peptide B thioester, the N-terminal residue Cys was protected by Npys after reaction with 2,2′-dithiobis(5-nitropyridin) for facilitating the subsequent thioacid capture ligation reaction. After 6 h, the reaction with 2,2′-dithiobis(5-nitropyridin) was completed. The product was purified by HPLC and the molecular weight was confirmed by ESI-MS (figure 3).

### 3.4. Solid-phase synthesis of peptide C

**Figure 4.**
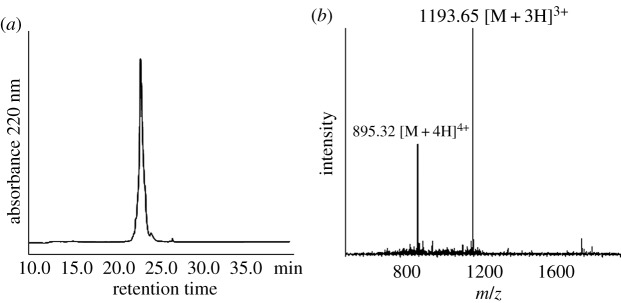
Characterization of peptide C. (*a*) C18 analytical HPLC profile of peptide C. HPLC condition: 0% to 20% of solvent B in solvent A in 5 min, followed by 20%–60% of solvent B in solvent A in 40 min. (*b*) Mass spectrum of this peptide determined by ESI-MS. [M + 3H]^3+^ found: 1193.65, MW calcd: 3577.2.

Standard Fmoc SPPS was used for the preparation of peptide C, with the sequence of H-**CSDKLFRADISEDYKTRGRKLLRFNGPV-PPP**-OH. The C-terminal ester bond was generated on the Wang resin using DIC as the coupling reagent overnight. After cleavage and purification, the pure peptide C was obtained as shown in figure 4.

### 3.5. Ligation of peptide A thioacid and Npys-peptide B thioester

**Figure 5.**
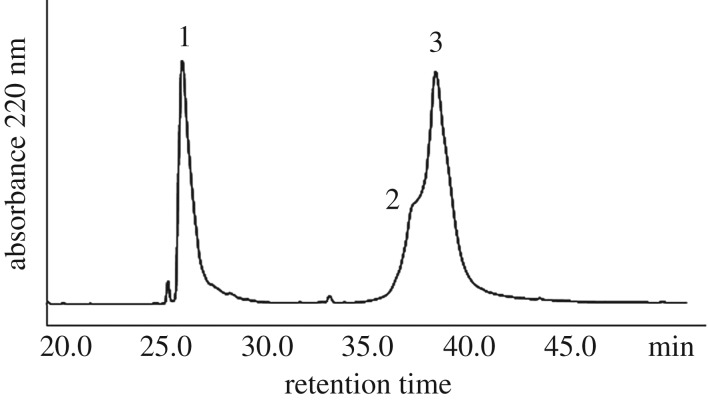
C18 analytical HPLC analysis of the ligation reaction. HPLC condition: 0% to 20% of solvent B in solvent A in 5 min, followed by 20%–60% of solvent B in solvent A in 40 min. Solvent A: 0.045% TFA in H_2_O, solvent B: 90% acetonitrile in H_2_O. Peak 1 is peptide B thioester, peak 2 is peptide A thioacid and peak 3 is the ligation product.

**Figure 6.**
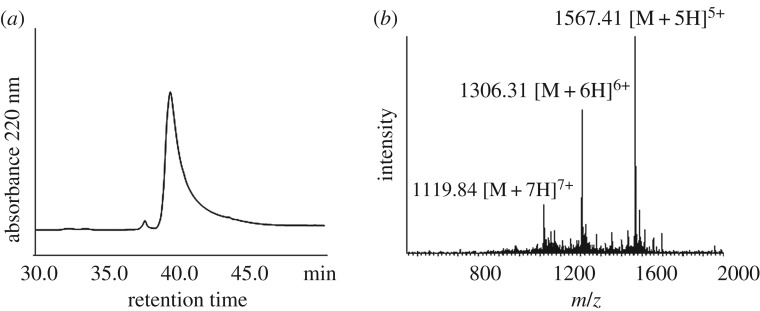
Characterization of peptide A + B. (*a*) C18 analytical HPLC profile of peptide A + B. HPLC condition: 0% to 20% of solvent B in solvent A in 5 min, followed by 20%–60% of solvent B in solvent A in 40 min. (*b*) Mass spectrum of this peptide determined by ESI-MS. [M + 5H]^5+^ found: 1567.41, MW calcd: 7831.9.

The thioacid capture ligation is efficient. The capture step is instantaneous because of the activated Npys-S disulfide and the supernucleophilicity and low p*K*_a_ value of the thioacid compared to a normal alkyl sulfhydryl group. The intramolecular acyl transfer step is also rapid, as the acyl disulfide activates the carbonyl group for a highly efficient nucleophilic substitution reaction with the closely positioned N^α^-amine. The capture reaction was carried out under weakly acidic condition, pH 2–3. After adjusting the pH to 6, the ligation reaction was allowed to proceed for 2 h at 37°C for a complete intramolecular acylation. Peptide A thioacid (4.6 mg, 5 mM) and Npys-peptide B thioester (6.8 mg, 10 mM) were the substrate. After incubation for 2 h, TCEP (final concentration 50 mM) was added to give a reductive environment. From the HPLC analysis, approximately 80% peptide A was ligated to peptide B (figure 5). No side reaction was detected. Since the C-terminal residue of peptide A thioacid is Pro, the ligation reaction took a relatively long time as compared to other ligation reactions with different C-terminal residues. However, it is remarkable that ligation at Pro-Cys can be achieved here. It is known from previous studies that it is practically impossible to ligate at Pro-Cys junction by the conventional native chemical ligation method. After purification by semi-prep and lyophilization, 4.7 mg purified ligation product (isolated yield 60%) was obtained (figure 6).

### 3.6. Ligation of peptide A + B thioester and peptide C

**Figure 7.**
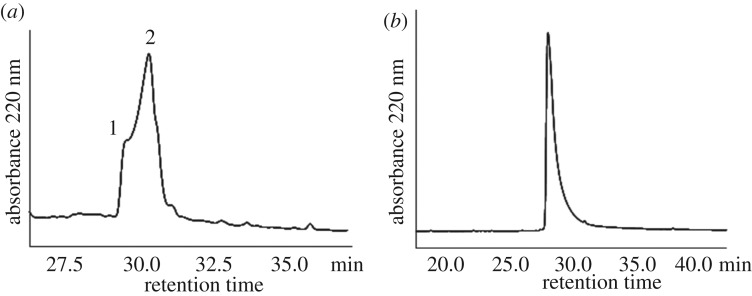
(*a*) C18 analytical HPLC analysis of the ligation reaction. HPLC condition: 0% to 50% of solvent B in solvent A in 20 min, followed by 50%–60% of solvent B in solvent A in 20 min. Peak 1 is peptide A + B thioester, and peak 2 is the ligation product. (*b*) C18 analytical HPLC profile of single-chain monellin. HPLC condition was the same as (*a*).

**Figure 8.**
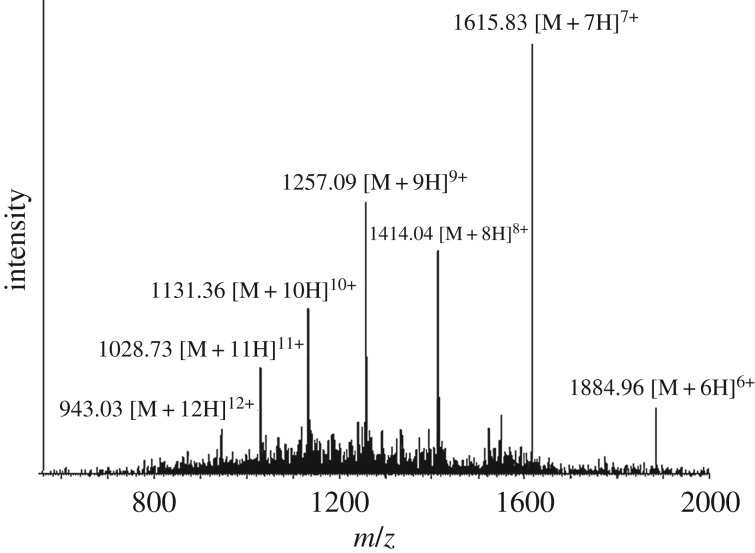
Mass spectrum of single chain monellin determined by ESI-MS. [M + 7H]^7+^ found: 1615.83, MW calcd: 11304.0.

**Figure 9.**
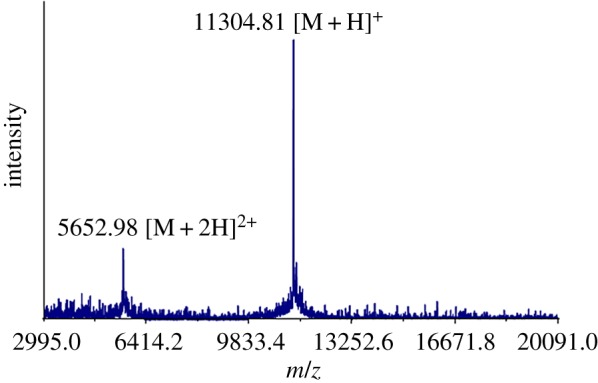
Mass spectrum of single chain monellin determined by MALDI-MS. [M + H]^+^ found: 11304.81, MW calcd: 11304.0.

We ligated peptide A + B thioester (4.7 mg, 5 mM) and peptide C (4.2 mg, 10 mM) by native chemical ligation. After two days reaction, the ligation yield can be reached to approximately 75% on HPLC analysis (figure 7*a*, peak 2). The ligation product, which represented the whole sequence of single-chain monellin, was purified by C18 analytical HPLC (figure 7*b*). The molecular weight of the synthetic monellin was confirmed by ESI-MS (figure 8) and MALDI-MS (figure 9) also. Finally, we got around 3 mg purified single chain monellin protein (isolated yield 44%).

### 3.7. Circular dichroism of the synthetic monellin

**Figure 10.**
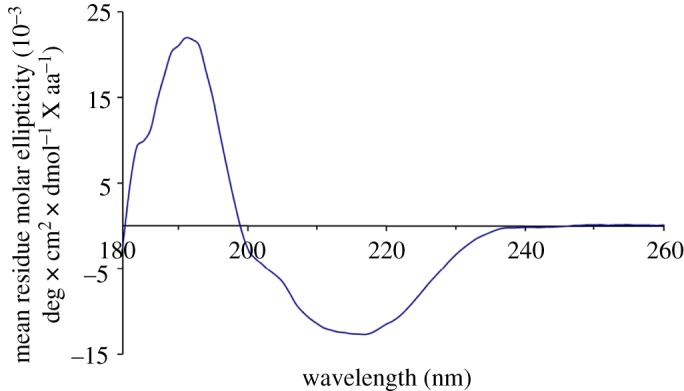
CD spectrum of the synthetic single-chain monellin.

The CD spectrum of the single-chain monellin was the same as that reported by Kim *et al.* [38] (figure 10). The sample was dissolved in 50 mM phosphate buffer (pH 7) at the concentration of 2.2 mg ml^−1^. The CD spectrum was recorded at 25°C. Monellin has a secondary structure consisting of five β-strands that form an anti-parallel β-sheet which has a positive absorption at 195 nm and a negative absorption at 215–217 nm, and an α-helix which has a positive absorption at 190 nm and a negative absorption at 208 nm and 222 nm in the CD spectrum. The replacement of the Ala to Cys did not affect the structure of monellin.

## 4. Conclusion

In summary, the total synthesis of single-chain monellin clearly demonstrates that one can combine thioacid capture ligation with native chemical ligation in the N-to-C sequential manner to synthesize a fully active protein. In principle, the thioacid capture ligation step can be repeated to allow multiple sequential ligations, since the thioester linkage of any intermediate ligation product can be converted to a thioacid. This work shows a new sequential ligation method of the two ligation chemistries as well as the versatility of a thioacid or thioester group in protein synthesis.

## Data accessibility

This article has no additional data.
